# Cannabinoids in Medical Practice

**DOI:** 10.1089/can.2015.0010

**Published:** 2016-01-01

**Authors:** Thomas B. Strouse

**Affiliations:** Department of Psychiatry, David Geffen UCLA School of Medicine, Los Angeles, California.

**Keywords:** cannabinoids, efficacy of cannabinoids, medical marijuana, physician prescribing of cannabinoids, risks of cannabinoids

## Abstract

Many patients with chronic medical illnesses use cannabinoids. There are two FDA-approved cannabinoid products, whereas medical marijuana purchased at legal dispensaries is not FDA regulated and may contain uncertain concentrations of various compounds. Cannabinoids have shown efficacy in treating chemotherapy-related nausea and vomiting, poor appetite in advanced HIV, some pain states, and multiple sclerosis-associated spasticity. Recreational cannabinoid use has many known potential serious harms. Physicians should be knowledgeable about cannabinoids and should inquire with their patients about cannabinoid use. Practical suggestions for clinical approaches are included.

I last editorialized on clinical uses of cannabinoids for medically ill persons in early 2015.^1^ At that time, I attempted to provide commentary on evidence-based uses of cannabinoids in the setting I know best: palliative care and cancer treatment.

Now, the editors of *Cannabis and Cannabinoid Research* have invited me to help provide readers with an update on the state of the evidence for the uses of cannabinoids in general clinical medicine. While an in-depth review is not possible in perspective format, some important general points can be made.

Where might this important discussion start?

Much of contemporary medical practice remains palliative—that is to say, physicians and other healers often see and treat patients with chronic burdensome conditions unlikely to be cured. Many of those patients suffer from symptoms that are not fixed by either disease-modifying or standard palliative treatments.

It makes sense then that patients with such conditions might choose—with or without the involvement of a physician responsible for their longitudinal care—to explore a range of alternative/integrative options, including medical marijuana, for symptom management or other efforts to improve disease-related quality of life. What is the state of the science regarding cannabinoids for symptom management in medical illness? How should we be counseling our patients? What kind of inferences can be drawn by clinicians in the service of trying to help our patients translate from the available clinical science to the bedside and to the counter at the marijuana dispensary? What possible jeopardy awaits physicians who endorse medical marijuana use by their patients?

## Definition of Terms

The marijuana plant contains many cannabinoid molecules, although THC and CBD are the most widely studied and have differing pharmacologic properties. There is also wide variation of THC and CBD concentrations both within specimens of the same marijuana strains and among different strains. In this commentary, marijuana, medical marijuana, and cannabis refer to naturally grown plant materials that are not approved or regulated by the FDA, and which are procured by patients in a variety of forms (edible, drinkable, volatile) from legal marijuana dispensaries or street suppliers. Cannabinoids refer to three chemical classes of compounds: naturally occurring molecules found in the cannabis plant, synthesized molecules, and the so-called endocannabinoids, which are produced in the central nervous system (CNS) of most animals. Pharmaceutical cannabinoids refer to those cannabinoids that have demonstrated safety and efficacy to treat specific clinical problems and have been approved by a national regulatory agency such as the FDA for manufacture and sale based on a physician's prescription. In this latter circumstance, the companies that legally produce pharmaceutical cannabinoids are subject to the same manufacturing standards for safety/purity/content required by FDA (or its counterpart in other countries) for other drugs and devices.

## The Big Picture

In July 2014, New York became the 23rd state (plus Washington DC) to legalize personal marijuana possession and its consumption for putative medical purposes. A full-page New York Times advertising spread appeared a few days later, sponsored by the web app *Leafly* to kick off its Just Say Know campaign, congratulating the state on progressive action and endorsing the use of cannabis for medical symptom management. Utilizing pseudoscientific labeling that recalled the Periodic Table of the Elements, the advertisement featured Ian, who chose an Indica cannabis strain to relieve his multiple sclerosis (MS) symptoms, and Molly, who while fighting cancer preferred Sativa cannabis.^*^ No evidence was provided to support the claims that cannabis works for either indication. Perhaps the advertisers were confident that popular beliefs are sufficient.

The legalization of medical marijuana in New York State generated journalistic responses as well. The New York Times editorial board published a weeklong series of daily editorials—some of which called on the US government to repeal its ban on marijuana, which dates to the 1970 Controlled Substances Act. Most recently, this call was joined by US Senator and Democratic Presidential Candidate Bernie Sanders, who introduced in late October 2015 The Ending Federal Prohibition of Marijuana Act, a senate bill which would delegate regulatory authority over marijuana to the states in the manner of tobacco and alcohol.^2^

Most of the American public appears to be ready to legalize doctor-supervised medical marijuana: in a January 2014 ABC News poll, 86% favored legalization for seriously ill patients. As noted above, 23 states and Washington DC have passed medical marijuana laws intended to decriminalize possession for personal use or so-called legitimate medical uses, with more states on the way. Colorado, Washington, Alaska, Oregon, and Washington DC have gone further by legalizing sale and possession for personal recreational use. Yet, a recent article in *The New England Journal* warns that Big Marijuana is unlikely to be any more concerned with the public's health, or any less voracious in its business practices, than Big Tobacco was during the 19th and 20th centuries.^3^

## The Current Armamentarium

At the time of this writing, there are two FDA-approved cannabinoid drugs available for prescription in the United States: dronabinol, a synthetic THC compound, and nabilone, a semisynthetic analog of THC approximately 10 times more potent than dronabinol.^4^ Both are approved for chemotherapy-associated nausea and vomiting; dronabinol is also approved for HIV-associated anorexia/wasting, although the evidence for the latter indication is slim and the problem is much rarer since the advent of antiretroviral drugs. Both dronabinol and nabilone have been studied as possible treatments for other symptoms; although each has shown some efficacy as an adjuvant analgesic, the sedating and psychotropic properties of both agents tend to limit their utility.

Nabiximols, an oral spray that is an approximately racemic mixture of THC and CBD, is approved in Canada for opioid-resistant, treatment-refractory cancer pain and MS-associated spasticity and central pain, and in the United Kingdom, Spain, and New Zealand, for MS-associated spasticity. It is undergoing phase 3 trials in the United States for cancer pain and may soon be available here.

All other medical marijuana ingested by patients in the United States represents products unregulated by the FDA and therefore with uncertain chemical content.

## Efficacy

Studies of cannabinoid efficacy are heterogeneous: some have tested whole leaf marijuana, others specific/isolated phytocannabinoids (such as THC), some cannabinoid combinations (such as nabiximols, the 1:1 THC:CBD compound), and some, synthesized compounds such as nabilone. This heterogeneity makes meta-analysis more complicated and adds to the complexity of drawing clinical inferences and decision making (see the Reliability/Reproducibility of Effects section below).

A recent Cochrane-style meta-analysis^5^ assessed the quality of the evidence assessing the effectiveness of cannabinoids in the treatment of nausea and vomiting due to chemotherapy (CINV), appetite stimulation in HIV/AIDS, chronic pain, spasticity from MS or paraplegia, depression, anxiety, sleep problems, psychosis, glaucoma, or Tourette's syndrome. Seventy-nine randomized controlled trials were identified, involving 6462 patients. The authors concluded that there was moderate quality evidence to support the use of cannabinoids for chronic pain and spasticity. They described as low the quality of the evidence supporting the efficacy of cannabinoids for CINV, HIV-associated wasting, sleep problems, and Tourette's syndrome. This latter result is a particularly striking finding since it was that low quality evidence that was sufficient to achieve US FDA approval for dronabinol and nabilone cannabinoid pharmaceuticals. In that same issue of *JAMA*, a companion review offered a clear summary of practical clinical and legal considerations related to medical marijuana in the United States.^6^

Another recent review looked at indications for cannabinoids that appear with high frequency in various state regulations that outline qualifying conditions for medical marijuana.^7^ These include Alzheimer's disease, amyotrophic lateral sclerosis, cachexia, cancer, Crohn's/inflammatory bowel disease, epilepsy, severe/chronic pain, glaucoma, hepatitis C, HIV/AIDS, MS, and post-traumatic stress disorder. In this meta-analysis, only cachexia and pain were judged to be sufficiently supported by human trials to support credible evidence-based use. Among other things, this suggests the extent to which state medical marijuana use guidelines may be influenced by popular beliefs and/or political processes. Readers interested in state-by-state listings of endorsed medical marijuana uses can consult their state's marijuana legal code or go to www.leafly.com/news/health/qualifying-conditions-for-medical-marijuana-by-state.

The American Academy of Neurology also recently published a review; it concluded that cannabinoids, particularly nabiximols, may yield marginal benefit in patients with MS for spasticity, central pain, and urinary symptoms,^8^ but identified little utility in other neurologic conditions. Friedman and Devinsky^9^ provided a scholarly summary of the evidence for treatment of epilepsy with cannabinoids. While acknowledging pre-clinical and preliminary/anecdotal clinical data, they emphasize the importance of standard double-blind trials to help improve the state of knowledge. Another recent review concludes that marijuana has equivocal effects on generic sleep problems, with some small benefit incurred for pain patients with sleep disturbance.^10^

There is some emerging evidence in pain management that cannabinoids may contribute to reversing opioid-associated hyperalgesia,^11^ may work synergistically to allow lowered opioid dosing,^12^ and may have unique efficacy in the prevention and/or treatment of chemotherapy-induced peripheral neuropathic pain.^13^

## Safety

Nora Volkow and her colleagues from the National Institute on Drug Abuse (NIDA) recently published an excellent review on the health risks of recreational marijuana.^14^

Recreational use of cannabinoids is particularly dangerous for the developing brains of young people and for individuals with existing substance abuse problems and other mental illnesses. Regular use can hasten or unmask psychotic illnesses, and it has been associated with diminished IQ. Individuals who use cannabinoids chronically can develop addiction and physical dependence, and there is a well-described withdrawal syndrome. Chronic marijuana use is also associated with increased risk for dropping out of school, overall diminished life satisfaction and achievements, and chronic bronchitis. Short-term use of (presumably high THC-containing) recreational marijuana impairs memory, motor coordination, and judgment. All of these are highly concerning findings and may well apply equally to the regular medical marijuana user and the recreational user, who some believe differ only in the stated intent or use motive.

There is also concerning evidence about the public health consequences of widespread cannabis legalization. A recent study suggested that after cannabis legalization in Colorado, there was a two-fold increase in the frequency of marijuana-positive drivers in fatal auto crashes, with no increase in alcohol.^15^

Balanced against these individual and public safety concerns is a recent finding in state-by-state studies correlating significant reductions in opioid overdose deaths after the enactment of medical marijuana laws.^16^ While the nature of this correlation remains uncertain, it raises interesting public policy questions and calls out for further study.

I have argued elsewhere that the concerning safety findings outlined above may be less important for clinical decision-making in a patient with advanced or terminal medical illness who agrees not to drive automobiles or operate other potentially dangerous machinery under the influence. For middle-aged and older adults with foreshortened life expectancy due to medical illness, both the physician and the patient might conclude that the potential behavioral and intellectual toxicities of cannabinoids could tilt the risk/benefit scale in a different direction than for a younger patient with chronic, but not life-limiting, medical illness, who expects to try to compete in the workforce, operate heavy machinery in public venues, has parent-dependent children, and wants to learn new life and job skills.

The point of this comparison is to underscore that all other things being equal, the thoughtful consideration of the potential benefits of cannabinoids for a given patient requires the perspectives of the treater (if there is one), the patient, the family, the specific clinical circumstances, disease state and expected natural history, personal developmental state, life aspirations, and other considerations. Put differently, an optimal approach to this decision includes a careful risk/benefit discussion and continued close clinical oversight.

## Reliability/Reproducibility of Effects

There is at best inconsistent evidence that the emerging marijuana industry has made efforts to conduct quality assurance activities. Some dispensaries, for example, promise that they measure and warrant the chemical composition of each batch of their products.

A reasonable generalization regarding the current state of affairs, however, is that the cannabis our patients purchase at the local cooperative will likely contain uncertain concentrations of THC/CBD and other compounds, despite what the label says. For example, a recent small study of marijuana edibles (75 products randomly purchased from internet-listed dispensaries in San Francisco, Los Angeles, and Seattle) showed accurate labeling of THC/CBD content in only 17%.^17^ The majority (60%) were overlabeled, (at least 10% less cannabinoid content than claimed), while 23% were underlabeled (at least 10% more cannabinoid content than labeled). Interestingly, Los Angeles dispensaries, compared with the others, showed a propensity to underlabel (*p*=0.01).

These findings underscore the important reality often misunderstood by patients who may not be familiar with the rigors of FDA-approved pharmaceutical manufacturing that most discussions about medical marijuana as a kind of drug therapy represent a leap of faith that the patient has actually received what he/she thinks was purchased. In addition to undermining efforts at a discussion about the reliability and reproducibility of effects, this fact raises a host of other considerations, including basic safety (might there be the presence of adulterants, congeners, contaminants, insecticides), dose-related concerns (little or no pharmacologic effect at one end and drug-related toxicities at the other), and potentially differing pharmacologic effects from batch to batch, just to name a few. It also adds an additional level of uncertainty to any efforts by the clinician to consider/discuss/counsel patients about dose, drug–drug interactions, and other routine clinical issues that might arise around the prescription or endorsement of a new treatment. Route of administration raises additional levels of uncertainty: bioavailability varies significantly depending on whether cannabis is smoked, vaporized, or orally ingested.^18^

## Take Home

Some patients with difficult to manage symptoms may benefit from the utilization of cannabinoids. Public policy and law enforcement practices related to medical uses of cannabinoids should be governed by science.^19^ Since there is a paucity of clinical trial evidence for the superiority of cannabinoids (in any form) over approved drug therapies, it makes sense for physicians to agree to support their patients to trial medical marijuana when standard treatments are not helping enough. Cannabinoids may also be reasonable options when patients for personal reasons prefer them as first-line treatments over other standard treatments, although it is difficult to provide advice to physicians about how to participate in such activities.

In recommending an approach to determine which patients might be appropriate candidates for physician-endorsed cannabinoids, Hill^6^ identifies five commonsense criteria: (i) the patient should have a medical condition for which adequate clinical trials showed that cannabis has efficacy; (ii) the patient should have failed first- and second-line noncannabinoid pharmacotherapies; (iii) the patient should have failed an FDA-approved cannabinoid (dronabinol or nabilone); (iv) the patient should have no known substance abuse or psychotic illness and no unstable primary mood or anxiety disorder; and (v) the patient should live in a state where medical marijuana is legal. Beyond Hill's commonsense suggestions, I offer the following addenda:

1. There is reasonably good evidence that cannabinoids may help for some forms of chronic (particularly neuropathic) pain and MS-related spasticity. Cannabinoid use is also accepted in the treatment of CINV and cachexia associated with cancer and HIV, although the science in support of these uses is not very strong. The evidence that cannabinoids may help for other symptoms, such as sleep problems, depression, Tourette's syndrome, or refractory seizures, is much weaker.
2. There are FDA-approved cannabinoid prescription formulations that should probably be first-line agents in physician supervised cannabinoid trials. All parties will then know precisely the chemical composition of what is being ingested and there will be clear evidence that federally (FDA-approved) legal approaches were trialed, although as noted elsewhere utility of these agents is often limited by side effects. Physicians can *prescribe* these.
3. Nonprescribing actions by physicians related to non-FDA-approved medical marijuana vary by state, but are generally defined in the laws as *endorsements*, *attestations*, or *certifications* of the possible efficacy of medical marijuana for a particular problem or symptom. Not all physicians will be comfortable signing such a certification (see item 9 below). In California and many other states, there are physicians who are self-identified as being available to perform case reviews and to complete the attestation paperwork that allows patients to purchase a medical marijuana ID card, which is then used to gain admittance and to purchase from a dispensary.
4. For patients who wish to purchase/ingest medical marijuana with oversight by a continuity of care physician, the doctor should emphasize that there is a measure of uncertainty about whether the chemical composition of what they (patients) believe they are purchasing is in fact what they (patients) are being sold. This uncertainty also makes more speculative efforts to link patients' purchased medical marijuana products to published trial data and therefore to give standard, informed medical advice.
5. Clinicians should warn patients about the known risks associated with cannabinoids, with the goal of extending the conversation into a risk/benefit discussion. Risk review should extend to friends and family, including children, who may purposefully or inadvertently be exposed to the patients' medicine.
6. With these risks in mind, we should instruct patients to safeguard all cannabinoids as they would opioids, benzodiazepines, psychostimulants, and other controlled substances. In the absence of compelling data to the contrary, we should also be providing routine cautions to our patients as we would if they were starting on any other CNS-active medicine: care about walking and fall risk, care about operating automobiles and heavy machinery, and care about coadministration with other CNS-active agents, including alcohol.
7. Available evidence does not support a common cultural notion that cannabinoids as medicine are good for whatever ails you. Some people believe this to be true, however, and there is little doubt that in the unregulated world of the dispensary, all manner of health claims are being made. We probably need to consider and respond to such belief systems as we would other sociocultural views that seem to exist outside or beyond the available scientific evidence: respectfully, nonjudgmentally, and professionally. At the same time, we should make efforts to help our patients think critically about these kinds of assertions, particularly if they are leading our patients to eschew evidence-based disease-modifying treatments in ways that seem to be risky.
8. If we are going to endorse or tacitly support medical marijuana use by our patients, we need to develop basic competence in recognizing toxicities associated with it: intoxication, abuse, withdrawal states, and other side effects.
9. Where we encounter problematic use of cannabinoids—use that constitutes abuse or dependence—we should be prepared to identify the problem, explore its context and consequences, and advocate for appropriate treatment, just as we would for our patients who might develop similar problems with other prescribed substances or alcohol.
10. Physicians who wish to avoid finding themselves at the fulcrum between conflicting state and federal laws regarding marijuana (which is bafflingly still classified under Federal law as a Schedule I drug—no medical use and high abuse potential) should not participate as partners or shareholders in the rapidly expanding marijuana production and dispensary industry. It appears unlikely in this era that physicians would be harassed by the DEA for simply talking with their patients about potential beneficial uses of medical marijuana, or completing an attestation form, but in some states, physicians who have served as medical officers or dispensary board members have been intimidated by DEA agents.^20^

Some physicians who care for patients with chronic illnesses and associated significant symptom burden take a “don't ask, don't tell” position regarding medical marijuana. Despite its convenience and tidiness, this is an increasingly untenable position. Medical marijuana and cannabinoid pharmaceuticals seem to be here for the duration; there is a credible evidence base for their efficacy; they are widely available; and they are widely used. Conventional approved treatments are imperfect, and patients and families are often desperate to find alternatives.

I have come to see willful ignorance about cannabinoids as a form of patient abandonment. The message to the patient seems to be figure it out yourself and do not tell me about it. Such a stance is not consistent with the highest values and aspirations of medicine.

## Notes

^*^ Leafly, Inc. Advertisement in New York Times, August 3, 2014, p. A3.

## Abbreviations Used

CBD: cannabidiol
CNS: central nervous system
MS: multiple sclerosis
NIDA: National Institute on Drug Abuse
THC: tetrahydrocannabinol
